# Investigating antibacterial and anti-inflammatory properties of synthetic curcuminoids

**DOI:** 10.3389/fmed.2024.1478122

**Published:** 2024-10-29

**Authors:** Kateřina Veselá, Zdeněk Kejík, Nikita Abramenko, Robert Kaplánek, Milan Jakubek, Jitka Petrlova

**Affiliations:** ^1^BIOCEV, Biotechnology and Biomedicine Center of the Academy of Sciences and Charles University in Vestec, Vestec, Czechia; ^2^Department of Paediatrics and Inherited Metabolic Disorders, First Faculty of Medicine, Charles University and General University Hospital in Prague, Prague, Czechia; ^3^Department of Biomedical Science, Faculty of Health and Society, Malmö University, Malmö, Sweden

**Keywords:** curcuminoids, antibacterial, anti-inflammatory, immunomodulatory, lipopolysaccharide

## Abstract

The concept of intratumoral microbiota is gaining attention in current research. Tumor-associated microbiota can activate oncogenic signaling pathways such as NF-κB, thereby promoting tumor development and progression. Numerous studies have demonstrated that curcumin and its analogs possess strong antitumor effects by targeting the NF-κB signaling pathway, along with potent antibacterial properties. In this study, we tested the antibacterial activity of two curcuminoids, Py-cPen and V-cPen, against the Gram-negative bacterial strains *Pseudomonas aeruginosa* and *Escherichia coli* and the Gram-positive bacterial strain *Streptococcus aureus* using *in vitro* assays and fluorescent microscopy. We observed that both Py-cPen and V-cPen reduced NF-κB activation upon lipopolysacharide (LPS) challenge in cell assays. In addition, our findings indicate that Py-cPen and V-cPen interact with LPS, as demonstrated by transmission electron microscopy and confirmed using *in silico* analyses, thereby modulating LPS activity. Overall, our data indicate that Py-cPen and V-cPen exhibit strong antibacterial and antiinflammatory properties, suggesting their potential as candidates for new multitarget therapeutic strategies.

## Graphical abstract

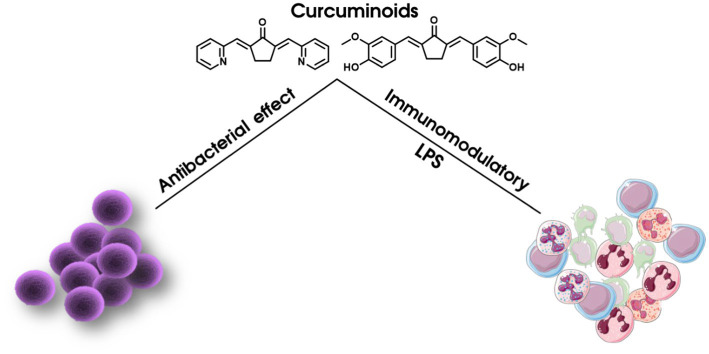

## 1 Introduction

Curcumin, a natural yellow polyphenol isolated from the rhizomes of *Curcuma longa*, has a range of beneficial effects, including anti-inflammatory, antibacterial, wound healing, and antioxidant or anticancer properties (1, 2). Curcumin has broad-spectrum antibacterial effects against a wide range of Gram-positive and Gram-negative bacteria (3, 4). The intratumoral microbiota is an important part of the tumor microenvironment. Microorganisms, which include viruses, fungi, and bacteria, have an important role in the human body. Intratumoral microbes (such as *Staphylococcus*) can promote tumor initiation and progression, e.g., by promoting inflammatory responses through activation of the nuclear factor kappa-B (NF-κB) pathway (5, 6). This activation can create a positive feedback loop, induce pro-inflammatory responses, and promote tumor progression, leading to a chronic inflammatory state that further accelerates tumorigenesis (7, 8). Recent studies suggest that intratumoral microorganisms may initiate tumor metastasis (9, 10). Specifically, *Staphylococcus* and *Streptococcus* in breast cancer tumor cells, e.g., have led to a reorganization of the cytoskeleton, which helps tumor cells overcome mechanical stress in blood vessels and promotes metastasis (9). Curcumin has also been found to be effective against microorganisms that are responsible for surgical infections, primarily *Staphylococcus aureus* and *Escherichia coli* (3). The antibacterial effects of curcumin include, e.g., disruption of the bacterial membrane, inhibition of the production of bacterial virulence factors and biofilm formation, and induction of oxidative stress (11, 12).

The anti-inflammatory effect of curcumin is attributed to the regulation of inflammatory signaling pathways and inhibition of inflammatory mediator production. Curcumin binds to toll-like receptors (TLRs), regulates the downstream of NF-κB, and exhibits both anti-inflammatory and anticancer properties (13, 14). The NF-κB pathway can be activated not only by numerous inflammatory stimuli including cytokines [e.g., interleukin 6 (IL-6) and tumor necrosis factor-alpha (TNF-*α*)] (15) but also by pathogen-derived molecules such as lipopolysaccharide (LPS). LPS is a component of the wall of Gram-negative bacteria and is recognized by TLR4 (14, 16, 17).

Curcumin is described to interfere with LPS-induced inflammatory pathways, including binding to cell surface receptors of LPS. Meng et al. found that curcumin suppressed LPS-induced overexpression of inflammatory mediators in rat vascular smooth muscle cells *in vitro*. Curcumin inhibited the overexpression of monocyte chemoattractant protein 1 (MCP-1), TNF-*α*, and nitric oxide (NO). They further found that curcumin inhibited the inflammatory response by suppressing TLR4 and NF-κB activation, inhibiting extracellular signal-regulated kinases 1/2 (ERK1/2) and p38 mitogen-activated protein kinases (MAPK), and reducing NADPH-mediated reactive oxygen species (ROS) (18).

Curcumin as a lipophilic agent is insoluble in water, which limits its bioavailability. Other limitations of curcumin are low absorption from the intestine, rapid metabolism, and rapid systemic elimination. There are several mechanisms to increase the bioavailability of curcumin, either by appropriate formulation or by preparation of new synthetic derivatives (19–21). Curcumin analogs are usually designed to exhibit several effects on biological targets such as a combination of anti-inflammatory, antimicrobial, and anticancer activities. The target could be, e.g., the NF-κB pathway, which is involved in tumor cell proliferation, apoptosis and angiogenesis, and inflammation (22).

Several curcumin analogs have been synthesized targeting the NF-κB signaling pathway. The focus of the structural analogs was mainly on heterocyclic analogs (23), cyclized pyrazole analogs (24), and the addition of methoxy groups or conjugated double bonds (25).

In this study, we explored the antibacterial and anti-inflammatory properties of two synthetic curcumin analogs, Py-cPen and V-cPen. Both compounds demonstrated efficacy against Gram-negative bacteria (*E. coli* and *P. aeruginosa*) and Gram-positive bacteria (*S. aureus*). In addition, our findings indicate that Py-cPen and V-cPen interact with LPS, which may account for their immunomodulatory effects on NF-κB activation and LPS uptake by immune cells during the LPS challenge. The obtained results suggest that both Py-cPen and V-cPen derivatives show promising antibacterial and anti-inflammatory properties and can potentially be used as adjuvants.

## 2 Materials and methods

### 2.1 Bacterial strains and endotoxin

*Escherichia coli* (25922), *Pseudomonas aeruginosa* (15159), and *Staphylococcus aureus* (29213) were purchased from the American Type Culture Collection (ATCC).

LPS from *Escherichia coli* (serotype 0111:B4, cat#L3024) was purchased from Sigma-Aldrich.

### 2.2 Curcuminoids and peptide

**Figure 1:**
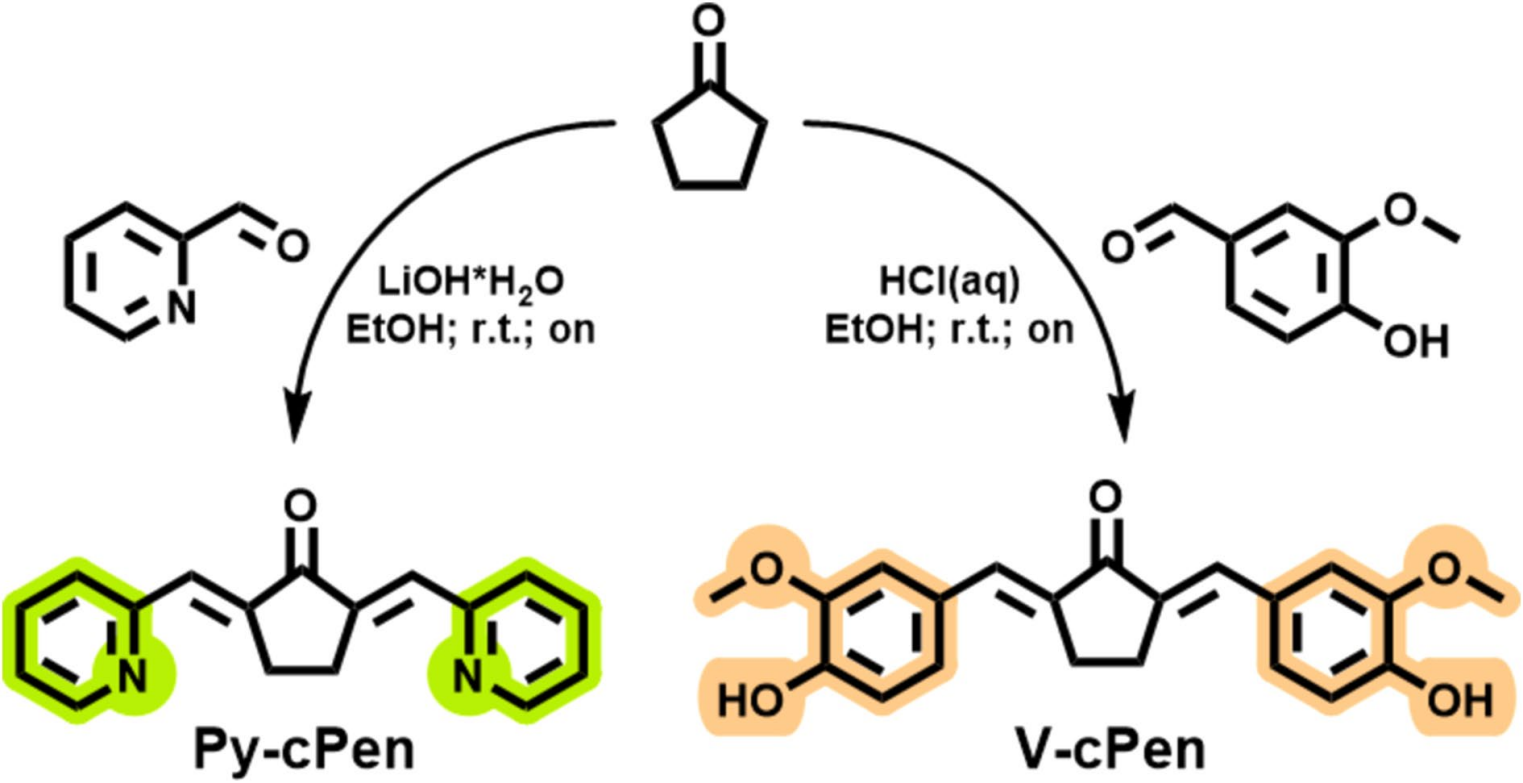
Scheme of synthesis and chemical structures of tested synthetic curcuminoids Py-cPen and V-cPen.

It was reported that the conjugated *β*-diketone causes instability and metabolic liabilities in natural curcumins; therefore, this substructure was replaced by a single ketone in the studied derivates (26). Two curcuminoids were designed as (2-pyridyl)methylidene and (4-hydroxy-3-methoxyphenyl)methylidene cyclopentanones (Figure 1). Curcuminoids (Py-cPen and V-cPen) were synthesized in the laboratory of the medicinal chemistry group of the First Medical Faculty of Charles University BIOCEV, Vestec, Czech Republic, following a prescribed synthesis (27, 28).

Cathelicidin antimicrobial peptide LL37 was purchased from Innovagen (United States) (29, 30).

### 2.3 Cells

THP-1-XBlue-CD14 reporter monocytes (InvivoGen, United States) were cultured in RPMI 1640-GlutaMAX-1 (Gibco, Life Technology Ltd., Renfrew, UK), and the media was supplemented with 10% (v/v) heat-inactivated FBS (FBSi, Invitrogen, Waltham, MA, United States) and 1% (v/v) antibiotic-antimycotic solution (AA, Invitrogen) at 37°C in 5% CO_2_.

RAW 264.7 (InvivoGen) cells were cultured in DMEM (HyClone, GE Healthcare Life Science, United States) supplemented with 10% (v/v) heat-inactivated FBS (FBSi; Invitrogen, United States) and 1% (v/v) antibiotic-antimycotic solution (AA; Invitrogen) at 37°C in 5% CO_2_.

### 2.4 Viable count assay

The potential antibacterial activity of curcuminoids (Py-cPen and V-cPen) on *E. coli*, *P. aeruginosa*, and *S. aureus* strains was explored by incubating one colony overnight in 5 mL of Todd-Hewitt (TH) medium. The next morning, the bacterial culture was refreshed and grown to a mid-logarithmic phase (OD_620nm_ = 0.4). The bacteria were then centrifuged, washed, and diluted 1:1,000 in 10 mM Tris buffer at pH 7.4 to obtain an approximate concentration of bacteria amounting to 2 × 10^6^ CFU/mL. Next, 50 μL of bacterial suspension was incubated with curcuminoids (Py-cPen 5, 10, and 30 μM and V-cPen 1, 3, and 10 μM), LL37 (1 μM) (used as a positive control), or a buffer control (10 mM Tris buffer at pH 7.4) for 2 h at 37°C. After 2 h, serial dilutions of the samples were plated on TH agar plates, incubated overnight at 37°C, and followed by colony counting the next day (31, 32).

### 2.5 Fluorescence microscopic analysis of LIVE/DEAD bacteria

The viability of *E. coli*, *P. aeruginosa,* and *S. aureus* was assessed using the LIVE/DEAD^®^ BacLight^™^ Bacterial Viability Kit (Invitrogen, Molecular Probes, Carlsbad, CA, United States). Bacterial suspensions were prepared for VCA as described above. Bacterial strains were treated with curcuminoids (Py-cPen 5, 10, and 30 μM and V-cPen 1, 3, and 10 μM) and LL37 (1 μM). After 2 h of incubation at 37°C, the samples were mixed 1:1 with the dye mixture, followed by incubation for 15 min in the dark at room temperature. The dye mixture was prepared according to the manufacturer’s protocol, i.e., 1.5 μL of component A (SYTO-9 green-fluorescent nucleic acid stain) and 1.5 μL of B (red-fluorescent nucleic acid stain propidium iodide) were dissolved in 1 mL of 10 mM Tris at pH 7.4. A measure of 5 μL of stained bacterial suspension was trapped between a slide and an 18-mm square coverslip. Overall, 10 view fields (1 × 1 mm) were examined from three independent sample preparations using a Zeiss AxioScope A.1 fluorescence microscope (objectives: Zeiss EC Plan-Neofluar 20×; camera: Zeiss AxioCam MRm; and acquisition software: Zeiss Zen 2.6 [blue edition]) (31, 32).

### 2.6 Molecular docking calculations

Molecular docking was performed using AutoDock Vina software. Only docking poses with a free energy of binding lower or approximately −5.5 kcal/mol were considered, which corresponds to an approximate K_i_ value of 0.1 mM or lower. The calculations were carried out using the parameters recommended in the user manual. AutoDock tools were used to find and determine the center and size of the grid box for the docking calculations.

The 3D structure of LPS was obtained by modeling two parts of a molecule from the PDB Data Bank using COOT software from the CCP4 package. The base part (lipid A) was obtained with ID 6BPP, and the O-antigen part was obtained with ID 7ML5. PDB IDs were downloaded, repaired, and saved, using the UCSF Chimera software, as .pdb file. The UCSF Chimera software was also used to remove unfavorable compounds such as water, solvents, and ligands so that only the molecule of LPS remained.

After that, both structures were joined using COOT software and edited using Avogadro software for the complete creation of bonds between O-antigen and lipid A parts.

The 3D structures of drugs (ligands) were obtained from the Jakubek’s Lab internal database. Atoms with double conformations were checked and repaired using a self-written script in Python programming language.

The predicted binding affinity (kcal/mol) was calculated by using AutoDock Vina software. To visualize molecules and analyze the docking results, two pieces of software were used. The PyMOL 3.0 software (by Schrödinger) was used to verify the positioning of the ligand on the receptor surface and to create a 3D structure of the complex. To generate overall views of the docking outcomes, UCSF Chimera was used, while BIOVIA Discovery Studio Visualizer (by Dassault Systèmes) was used to detect and illustrate the type of interactions of ligands (Py-cPen and V-cPen) with LPS.

### 2.7 Transmission electron microscopy

Curcuminoids were visualized using TEM (Jeol Jem 1,230; Jeol, Tokyo, Japan) in combination with negative staining after incubation with LPS or buffer. Images of endotoxin LPS (10 μM) in the presence or absence of Py-cPen (10 μM) or V-cPen (3 μM) were taken after incubation for 30 min at RT. For the mounted samples, 10 view fields were examined on the grid (pitch 62 μm) from three independent sample preparations. The samples were adsorbed onto carbon-coated grids (copper mesh, 400) for 60 s and stained with 7 μL of 2% uranyl acetate for 30 s. The grids were rendered hydrophilic via glow discharge at low air pressure. The size of LPS was analyzed as the mean gray value/μm^2^ ± SEM using an external software ImageJ 1.52 k. All images were converted to 8-bit (grayscale), and the threshold was adjusted accordingly (31).

### 2.8 NF-κB activity assay

NF-κB/AP-1 activation in THP-1-XBlue-CD14 reporter monocytes was determined after 20–24 h of incubation according to the manufacturer’s protocol (InvivoGen). Briefly, 1 × 10^6^ cells/mL in RPMI were seeded in 96-well plates (180 μL) and treated and incubated with curcuminoids (Py-cPen 10, 15, 20, and 30 μM and V-cPen 3, 5, 8, and 10 μM) or LL37 (1 μM), LPS (10 ng/mL) or both overnight at 37°C, 5% CO_2_ in a total volume of 200 μL. The following day, the activation of NF-κB/AP-1 was analyzed as the secretion of embryonic alkaline phosphatase (SEAP). The supernatant (20 μL) from the cells was transferred to 96-well plates, and 180 μL of Quanti-Blue was added. The plates were incubated for 2 h at 37°C, and the absorbance was measured at 600 nm using a VICTOR3 Multilabel Plate Counter spectrofluorometer (17, 31).

### 2.9 MTT viability assay

A volume of 20 μL of sterile filtered MTT solution (3-(4,5-dimethylthiazolyl)-2,5-diphenyltetrazolium bromide; Sigma-Aldrich) at 5 mg/mL in PBS was added to the remaining overnight culture of THP-1-XBlue-CD14 reporter monocytes from the above NF-κB activity assay in 96-well plates, which were incubated at 37°C (see above). After 2 h of incubation at 37°C, the supernatant was removed and the blue formazan product generated in cells was dissolved by adding 100 μL of DMSO to each well. The plates were then gently shaken for 10 min at room temperature to dissolve the precipitates. The absorbance was measured at 550 nm using a VICTOR3 Multilabel Plate Counter spectrofluorometer.

### 2.10 Phagocytosis assay

The macrophage cell line RAW 264.7 (passage 4–7) in DMEM was seeded in 96-well tissue culture plates (8 × 10^4^ cells per well) overnight at 37°C in a 5% CO_2_ atmosphere. Curcuminoids (Py-cPen 1, 5, 10, and 15 μM and V-cPen 1, 1.5, 3, and 5 μM) or LL37 (1 μM) were mixed with and without FITC-LPS (100 ng/mL). The pre-mixed samples (20 μL) were added to the adherent RAW 264.7 cells. To measure phagocytosis, the samples were incubated with the cells for 1 h at 37°C and washed twice using DMEM media. Then, fluorescence was measured using a VICTOR3 Multilabel Plate Counter spectrofluorometer (PerkinElmer, United States) at excitation/emission wavelengths of 530/570 nm. The baseline uptake (of only media) was subtracted from the signal of each sample. For analysis using fluorescence microscopy, RAW 264.7 (passage 4–7) cells were seeded in 24-well tissue culture plates with inserted coverslips (2 × 105 cells per well) overnight at 37°C in a 5% CO_2_ atmosphere. Then, the cells were treated as described above. After 1-h incubation at 37°C, the cells were stained with 300 ng/mL DAPI (Sigma-Aldrich, United States) for 3 min at room temperature, washed with PBS, and mounted onto glass slides using a mounting medium (Mountant, PermaFluor, Thermo Fisher Scientific, KU). In total, 10 view fields (1 × 1 mm) were examined from three independent sample preparations using a Zeiss AxioScope A.1 fluorescence microscope (objectives: Zeiss EC Plan-Neofluar 20×; camera: Zeiss AxioCam MRm; acquisition software: Zeiss Zen 2.6 [blue edition]) (17).

### 2.11 Statistical analysis

The graphs of VCA, TEM analysis, NF-κB activity, and phagocytosis are presented as the mean ± SEM from at least four independent experiments. Differences in these assays were assessed using a one-way ANOVA with Dunnett’s multiple comparison tests. All data were analyzed using GraphPad Prism 10 (GraphPad Software, Inc., La Jolla, CA, United States). In addition, *p*-values less than 0.05 were considered to be statistically significant (ns = not significant, *p < 0.05, **p < 0.01, ***p < 0.001, and ****p < 0.0001).

## 3 Results

### 3.1 Antibacterial activity of curcuminoids *in vitro*

**Figure 2:**
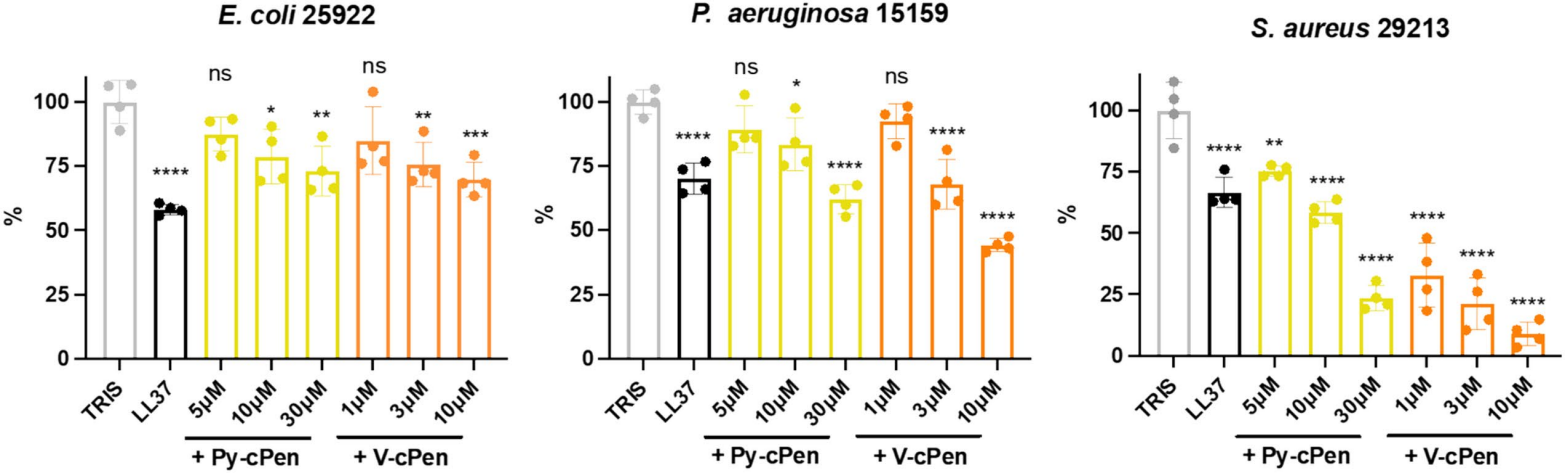
Antimicrobial activity of Py-cPen and V-cPen *in vitro*. VCA was used to analyze the antibacterial effects of Py-cPen (5, 10, and 30 μM) and V-cPen (1, 3, 10, and μM). LL37 (1 μM) was used as a positive control, and untreated (using TRIS) bacteria were used as a negative control. The concentration of bacteria (*E. coli*, *P. aeruginosa*, and *S. aureus*) was 2 × 10^6^ CFU/mL, and each sample was treated for 2 h. Data are presented as the mean ± SEM of four independent experiments (*n* = 4). Statistical analysis was performed using a one-way ANOVA with Dunnett’s multiple comparison tests. Results are reported as ns = not significant, *p < 0.05, **p < 0.01, ***p < 0.001, and ****p < 0.0001.

The antibacterial activity of curcuminoids Py-cPen and V-cPen against bacterial strains *of E. coli* (25922), *P. aeruginosa* (15159), and *S. aureus* (29213) was analyzed using VCA (Figure 2).

A bacterial growth reduction was observed with the application of both curcuminoid compounds. Py-cPen significantly decreased growth in all bacterial strains at concentrations of 10 and 30 μM. For Gram-negative strains such as *E. coli* and *P. aeruginosa*, Py-cPen at 10 μM caused approximately a 20% reduction, while at 30 μM, it led to a 30% reduction in growth for both strains.

V-cPen at 3 μM resulted in approximately a 30% reduction in both Gram-negative strains. At 10 μM, V-cPen significantly reduced growth by approximately 40% in *E. coli* and 60% in *P. aeruginosa*.

For the Gram-positive strain *S. aureus*, both curcuminoids caused a notable decrease in growth. Py-cPen led to nearly a 50% reduction at 10 μM, while V-cPen achieved an approximately 80% reduction at just 3 μM. TH agar plates illustrated a decline in colony numbers after treatment with Py-cPen and V-cPen across all bacterial strains (Supplementary Figures S1A–C). Cathelicidin antimicrobial peptide LL37 was used as a positive control to compare the effects of bacterial killing efficiency.

**Figure 3:**
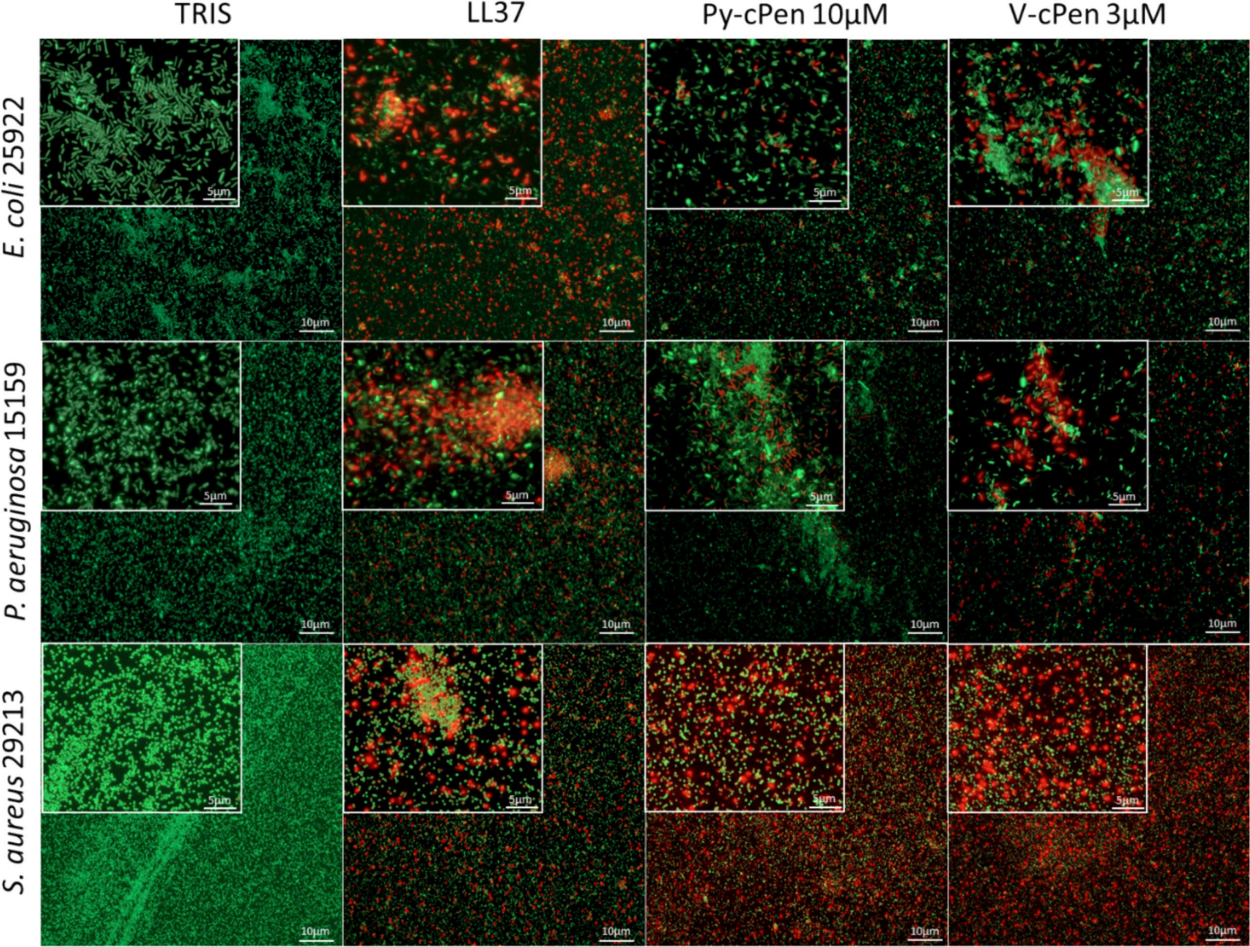
Visualization of *E. coli*, *P. aeruginosa*, and *S. aureus* viability. LIVE/DEAD Viability Assay of Gram-negative and Gram-positive bacteria stimulated with Py-cPen (10 μM) or V-cPen (3 μM), 10 mM Tris buffer at pH 7.4 as a negative control and LL37 (1 μM) as a positive control. Representative images for each independent experiment are presented (*n* = 4). Live bacteria were stained with green SYTO 9 nucleic acid fluorescent dye, and dead bacteria were stained with red propidium iodine dye.

The VCA data were confirmed using fluorescence microscopy with LIVE/DEAD staining (Figure 3). Dead bacteria with compromised membrane integrity were stained red, and live bacteria with intact membranes were stained green. The results indicated the presence of dead bacteria following treatment with 10 μM of Py-cPen and 3 μM of V-cPen. Additional experiments were conducted with Py-cPen at concentrations of 5 and 30 μM and using V-cPen at 1 and 10 μM. The data revealed a similar trend to that observed with VCA, showing an increase in killing ability with higher concentrations of both compounds (Supplementary Figures S2A,B).

### 3.2 Interaction of curcuminoids and LPS

**Figure 4:**
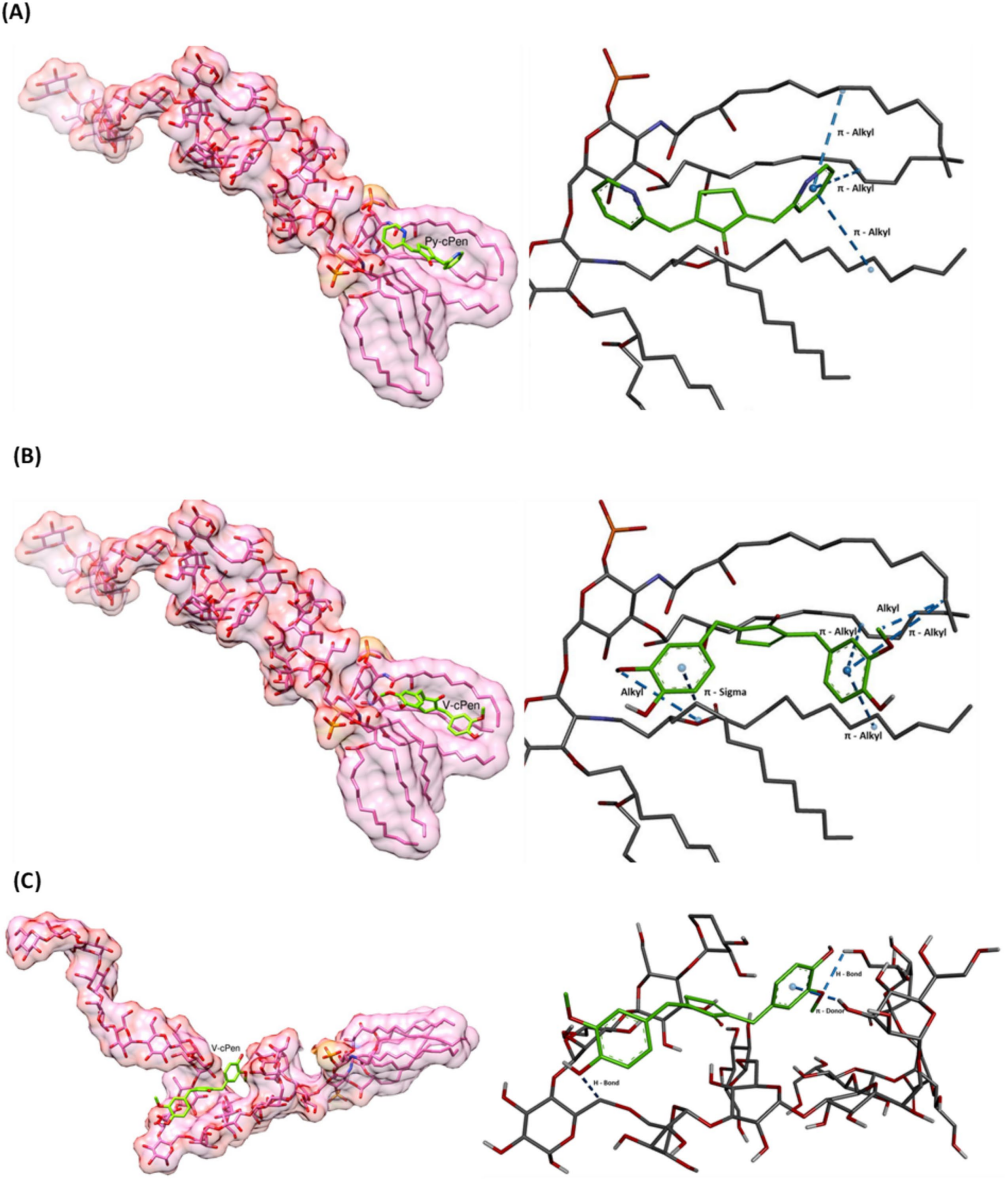
Molecular docking to investigate the binding affinity of **(A)** Py-cPen with lipid A part, **(B)** V-cPen with lipid A part, and **(C)** V-cPen with the part where core and o-antigen are joined parts of LPS. Both Py-cPen and V-cPen have shown favorable docking scores.

Molecular Docking with AutoDock Vina revealed that both curcuminoids (Py-cPen and V-cPen) exhibited favorable and comparable docking scores (−5.913 kcal/mol and −6.823 kcal/mol, respectively). This suggests their potential binding affinity for the aliphatic side chain of lipid A, which is a hydrophobic anchor and the most bioactive component of LPS (Figures 4A,B). It should be noted that V-cPen also showed high binding affinity in the part of LPS, where core and O-antigen are joined (−6.711 kcal/mol) (Figure 4C). This suggests that both curcuminoids have the potential to modulate LPS activity through their high binding activity. Thus, the results suggest that both Py-cPen and V-cPen exhibit a potential binding affinity to LPS, indicating their potential to modulate LPS activity.

Using BIOVIA Discovery Studio Visualizer, key interactions in the complexes of LPS with Py-cPen and V-cPen were detected. In the figures, the left side (generated using UCSF Chimera) displays the overall molecular structure, emphasizing the positioning of Py-cPen or V-cPen within the LPS molecule. Meanwhile, the right side (generated using BIOVIA Discovery Studio) provides a close-up view of these specific interactions.

As shown in Figure 4A, the visualization of Py-cPen reveals its interaction with the aliphatic side chain of the lipid A component of LPS. The primary interaction identified is a *π*-alkyl interaction, where the π-electron system of the pyridine group in Py-cPen engages with the aliphatic side chains of the glyceride. This hydrophobic interaction stabilizes the complex, while weak electrostatic forces between the π-cloud of the aromatic side group of curcumin and the glyceride alkyl chains contribute to the overall binding affinity (−5.913 kcal/mol). Notably, the *π*-alkyl interactions between the pyridine group of Py-cPen and the aliphatic chain of LPS are more centrally positioned within the glyceride region compared to V-cPen.

Figure 4B illustrates the interaction between the aromatic rings of guaiacol (ortho-methoxyphenol) in V-cPen and the aliphatic side chain of the lipid A portion of LPS, similar to the interactions observed with Py-cPen. However, V-cPen establishes additional interaction types with the lipid A component of LPS. In addition to *π*-alkyl interactions, V-cPen engages in π-sigma and alkyl interactions, enhancing its binding affinity to the hydrophobic region of lipid A (−6.823 kcal/mol) compared to Py-cPen. The π-sigma interaction occurs between the π-electron system of the guaiacol group in V-cPen and the *σ*-electron system of the aliphatic chain of lipid A. The π-alkyl interactions are situated more toward the periphery of the aliphatic region of the molecule, in contrast to the more central positioning observed with Py-cPen. The third type of interaction, alkyl, represents a hydrophobic interaction between the methoxy group of the ortho-methoxyphenol in V-cPen and the aliphatic side chain of lipid A.

Finally, Figure 4C depicts the affinity (−6.711 kcal/mol) between V-cPen and the polysaccharide part (O-antigen) of LPS. This visualization highlights three key interactions. A conventional hydrogen bond is formed between the oxygen atom of the methoxy group in the guaiacol side group of V-cPen and a hydrogen atom within the polysaccharide region of LPS. In addition, a carbon–hydrogen bond is observed, where the hydroxy group from the guaiacol group of V-cPen bonds with a carbon atom in the polysaccharide region. Finally, a *π*-donor hydrogen bond is established in which the π-electron cloud of the guaiacol acts as a donor for a hydrogen bond with the hydroxy group from the polysaccharide components of LPS.

One possible explanation for why Py-cPen and V-cPen are active at different concentrations is the interaction of V-cPen with two parts of the molecule LPS (lipid A and part where core and O-antigen are joined), whereas Py-cPen interacts only with the lipid A part of LPS.

**Figure 5:**
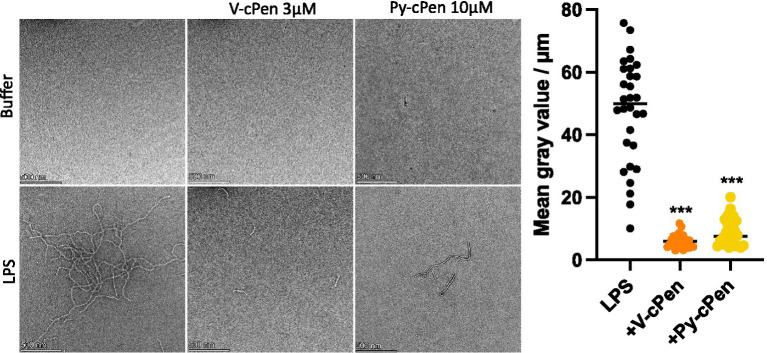
Py-cPen (10 μM) and V-cPen (3 μM) were incubated with LPS (100 μg/mL) for 30 min at RT and then presumable complexes were visualized by TEM. One representative image for each experiment is shown. Data are presented as the mean ± SEM of three independent experiments (*n* = 3). Statistical analysis was performed using a one-way ANOVA with Dunnett’s multiple comparison tests. Results are reported as ***p ≤ 0.001.

TEM analysis was performed to further examine the ability of curcuminoids to bind to LPS and alter its structural properties (Figure 5). The TEM images confirmed that the ribbon-like structure of LPS was significantly diminished upon the addition of Py-cPen (10 μM) and V-cPen (3 μM).

### 3.3 Immunomodulatory activity of curcuminoids *in vitro*

**Figure 6:**
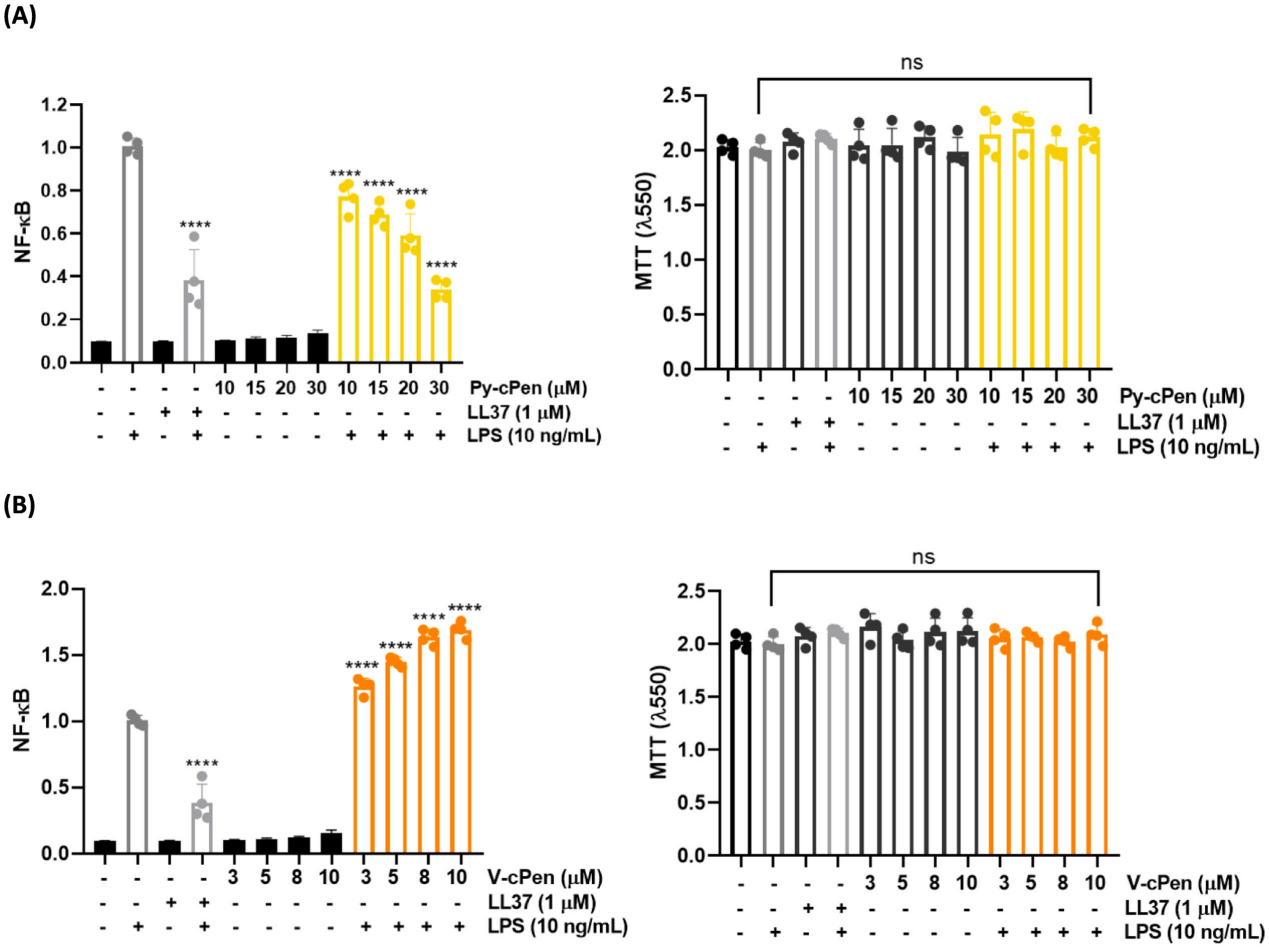
Inhibition of NF-κB activation using Py-cPen and V-cPen. **(A)** THP-1XBlue-CD14 cells were treated with Py-cPen (10–30 μM). In the case of Py-cPen, there is a significant reduction in the activation of NF-κB. **(B)** THP-1XBlue-CD14 cells were also treated with V-cPen (3–10 μM). V-cPen yielded the enhancement of NF-κB activation. LL37 (1 μM) was used as a positive control. The MTT viability assay was used to analyze the toxic effects of Py-cPen and V-cPen on THP-1XBlue-CD14 cells. Data are presented as the mean ± SEM of four independent experiments (*n* = 4). Statistical analysis was performed using a one-way ANOVA with Dunnett’s multiple comparison tests. Results are reported as ns = not significant and ****p < 0.0001.

We investigated the effect of curcuminoids (Py-cPen and V-cPen) on LPS signaling to determine NF-κB /AP-1 activation in THP-1XBlue-CD14 reporter monocytes (Figures 6A,B). Py-cPen (10–30 μM) inhibited NF-κB activation triggered by *E. coli* LPS. In contrast, V-cPen (3–10 μM) enhanced LPS-induced NF-κB activation. Neither of the curcuminoids showed a significant cytotoxic effect on THP-1XBlue-CD14, suggesting that the alteration of NF-κB activation was not due to toxic effects on the cells but to the effect of the curcuminoids themselves. LL37 peptide was used as a positive control.

**Figure 7:**
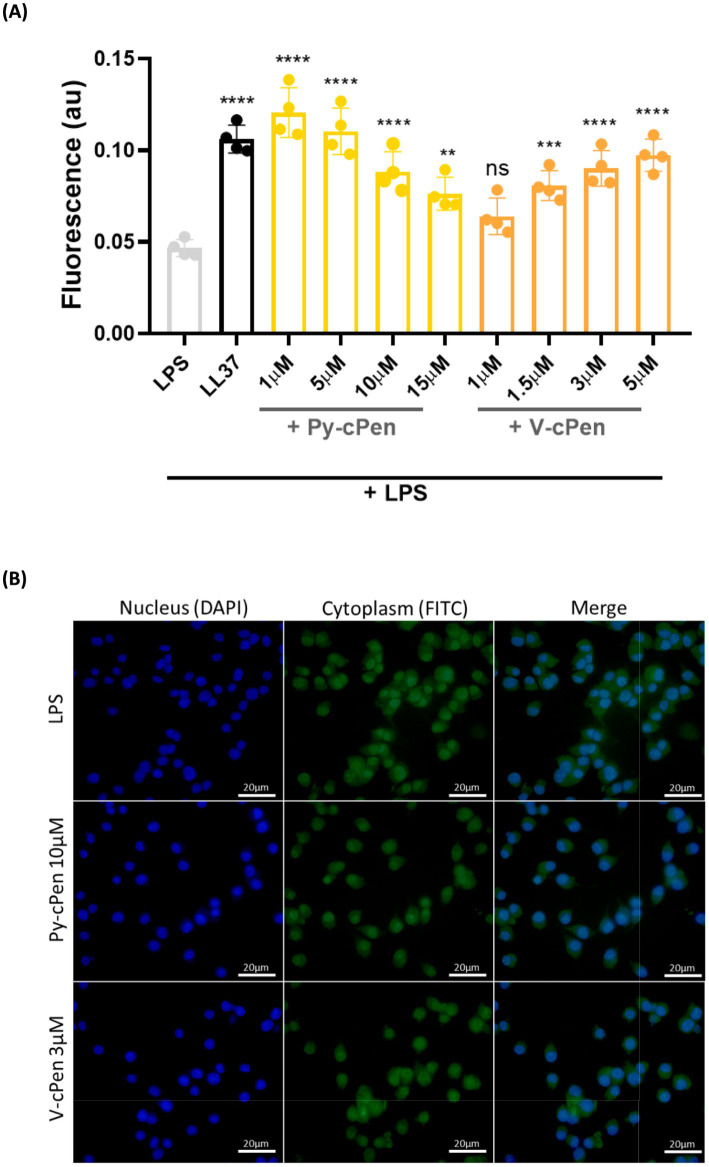
**(A)** Using the macrophage cell line RAW 264.7, a phagocytosis assay revealed a significant inhibition of translocation of NF-κB, stained with pre-treated LPS (FITC, green). **(B)** Fluorescence microscopy imaging translocation of NF-κB to the nucleus. Cytoplasm was stained with pre-treated LPS (FITC, green), and cell nuclei were visualized with DAPI (blue). RAW 264.7 cells were utilized for the experiment. Data are presented as the mean ± SEM of four independent experiments (*n* = 4). Statistical analysis was performed using a one-way ANOVA with Dunnett’s multiple comparison tests. Results are reported as ns = not significant, **p < 0.01, ***p < 0.001, and ****p < 0.0001.

Furthermore, a phagocytosis assay using fluorescence detection was conducted to investigate the effect of curcuminoids on uptake by RAW 264.7 cells (Figure 7B). RAW 264.7 cells were treated with Py-cPen (1–15 μM) and V-cPen (1–5 μM) or their mixtures with LPS (100 ng/mL). Both Py-cPen (1–15 μM) and V-cPen (1.5–5 μM) significantly increased FITC-LPS uptake by RAW 264.7 cells. LL37 was used as a positive control (reference). To confirm the uptake of LPS by RAW 264.7 cells, fluorescence microscopy imaging of cells stained with DAPI and FITC-LPS was performed (Figure 7A). RAW 264.7 cells were treated as described above but used only one concentration for Py-cPen (10 μM) and V-cPen (3 μM). This technique enabled the visualization of cells stained with DAPI and LPS labeled with FITC.

## 4 Discussion

In this study, we investigated the antibacterial and anti-inflammatory properties of synthetic curcuminoids. Curcumin, a natural bioactive substance, exhibits antibacterial, antioxidant, and anti-inflammatory effects. It has been found to have a higher sensitivity to Gram-positive than to Gram-negative bacteria (4).

Tyagi et al. investigated the antibacterial effect of curcumin against Gram-positive and Gram-negative bacteria and found, e.g., that 25 μM curcumin at a cell density of 10^6^ CFU/mL inhibited the growth of approximately 50% of *S. aureus* and approximately 20% of *E. coli* after 2 h of treatment (33).

Our synthesized curcuminoids showed excellent antibacterial effects against Gram-positive bacteria of *S. aureus* strain and also showed very good inhibitory effects against Gram-negative bacteria *E. coli* and *P. aeruginosa*. The curcuminoid V-cPen showed a significant inhibitory effect against *S. aureus* at a concentration of 1 μM, while Py-cPen was effective at a concentration of 5 μM. For Gram-negative bacteria, significant growth inhibition was observed with Py-cPen at 10 μM and V-cPen at 3 μM.

In the present time, the connection between microbial infiltration and tumor development has garnered increasing attention (5, 6, 34). For example, most of the patients with head-and-neck cancer experience inflammation of the oral mucosa after both radiotherapy and chemotherapy. The ulcer is colonized by bacteria (e.g., *S. aureus*, *E. coli*, or *P. aeruginosa*), which cause the release of endotoxins, further activating pro-inflammatory cytokines (35, 36). Curcumin, well known as a potent anticancer agent (37–39), has also been found to exhibit anti-inflammatory effects, by interacting with a component of Gram-negative bacteria LPS-induced inflammatory pathways such as NF-kB (40, 41).

It is important to note that *Staphylococcus aureus* is classified as a Gram-positive bacterium. In addition, lipoteichoic acid, a component of the *S. aureus* cell wall, has been shown to induce NF-κB signaling (42, 43). Reports indicate that the effects of curcumin against diseases induced by *S. aureus* are associated with the repression of NF-κB activation (44, 45). In addition, the potential synergy between lipopolysaccharides (LPS) and the inflammatory effects of *S. aureus* should not be overlooked. Zhang et al. have demonstrated that *S. aureus* bacteriophage can suppress LPS-induced inflammation in mammalian cells (46). In the context of tumor microbiota, interactions between microbial pathogens cannot be excluded. For instance, *P. aeruginosa*, when co-cultured with *S. aureus*, exhibits decreased sensitivity to *β*-lactam antibiotics, which is associated with alterations in membrane phospholipid composition (47). These findings suggest that the effects of curcumin on activated NF-κB signaling should be considered, even in the context of Gram-positive bacteria such as *S. aureus*.

As expected, Py-cPen demonstrated a reduction in NF-κB activation and inhibition of NF-κB nuclear translocation at a concentration as low as 10 μM. In the case of V-cPen, however, there was an increase in NF-κB activation. Based on these results, we determined LPS-induced NF-κB activation and nuclear translocation.

Similarly, Olivera et al. found that the EF31 analog was a more potent inhibitor of NF-κB activity (RAW 264.7) than curcumin, exhibiting both anti-inflammatory and anticancer activities (14). The structural motif of EF31 was also based on 2-pyridylmethyldiene, as was Py-cPen, confirming our results where Py-cPen was shown to inhibit NF-κB activity.

Nevertheless, curcumin regulation of NF-κB activity/signaling is a complex process. For example, Thota et al. reported that curcumin supplementation decreases circulating levels of glycogen synthase kinase-3*β* (GSK-3β) (48). Phosphorylation of NF-κB (Ser486) via GSK-3β can lead to decreased NF-κB activity (49). The curcumin effect on the transforming growth factor beta (TGF-β) signaling is associated with decreased peptidyl-prolyl cis/trans isomerase (Pin1) activity (50). Pin1 reduces p65 affinity to IkBα and supports stability and nuclear accumulation of p65 (51). Fumarate induces p65 phosphorylation (Ser536), leading to its activation and nuclear translocation via TANK-binding kinase-1 (TBK-1) (52). Ullah et al. reported that curcumin derivatives/analogs represent potential structure motives for the TBK-1 inhibitors (53).

The study by Bhattacharyya et al. suggested that the increased NF-κB activity and nuclear translocation after curcumin treatment may be due to its antioxidant properties, which liberate NF-κB from the inhibitory effects of induced ROS (54). Thus, it is possible that the increase in NF-κB activity after V-cPen treatment is due to its increased antioxidant activity, while NF-κB translocation is suppressed. Further studies are needed to determine the exact mechanism underlying this phenomenon.

The clinical implications of the obtained data warrant careful consideration. The tested curcuminoids demonstrate significant activity against *S. aureus*, a potential component of tumor-associated microbiota. For example, at a concentration of 1 μM, V-cPen reduces cell density to less than 50% of the original value. Notably, a more pronounced reduction in LPS stimulation is observed at a higher concentration of 3 μM. In the context of HL60 and K562 cells (28), the efficacy of V-cPen is occasionally diminished. In contrast, ATRA-resistant leukemic cells exhibit sensitivity comparable to that of *S. aureus*. It is important to highlight that the cytotoxic effects against lung fibroblasts are sometimes reduced, suggesting that the potential antitumor effects may be attributed not only to the direct targeting of tumor cells but also to the modulation of tumor microbiota and their associated carcinogenic effects. In the case of Py-cPen, a significantly higher efficiency in repressing LPS stimulation was observed, though at a slower rate compared to V-cPen, particularly in relation to the reduction of microbial populations. This observation aligns more closely with the anticancer effects of original curcumin (37). Moreover, Basak et al. reported that APG-157, a polyphenolic agent containing curcumin, reduces *Bacteroidetes* species in patients with oral cancer (55). These findings suggest that microbial targeting may play a crucial role in the anticancer effects of curcuminoids and should be more thoroughly considered in therapeutic strategies.

In this study, we performed *in silico* MD to investigate the potential interactions between the synthesized curcuminoids (Py-cPen and V-cPen) and LPS. The MD results suggest that both Py-cPen and V-cPen exhibit a potential binding affinity for LPS, indicating that these curcuminoids might modulate LPS activity. This interaction was further observed through TEM analysis, which showed a significant reduction in the LPS filaments after the addition of Py-cPen (10 μM) and V-cPen (3 μM). These findings suggest that the repressive effects of curcuminoids on bacteria-induced tumor inflammation could be associated with their direct interaction with bacterial LPS. In addition, these results should be considered in the design and interpretation of curcuminoid biological studies. However, further investigation is necessary to confirm this hypothesis.

## 5 Conclusion

The two synthetic curcuminoids, Py-cPen and V-cPen, exhibited antibacterial activity against both Gram-positive (*S. aureus*) and Gram-negative (*E. coli* and *P. aeruginosa*) bacteria. In addition, we confirmed the interaction between these synthetic curcuminoids and LPS, which may explain their anti-inflammatory effects due to a decrease in NF-κB activation and an increase in the phagocytosis of LPS. Thus, both the antibacterial and anti-inflammatory properties of these synthetic curcuminoids were investigated, suggesting that V-cPen and Py-cPen could be potential adjuvants in treating diseases associated with inflammation and bacterial contamination, such as head-and-neck cancer.

## Data Availability

The raw data supporting the conclusions of this article will be made available by the authors, without undue reservation.
