# Supplementary material for: Investigating antibacterial and anti-inflammatory properties of synthetic curcuminoids
Source: Front Med (Lausanne). 2024 Oct 29;11:1478122. doi: 10.3389/fmed.2024.1478122 (PMC11554473; doi:10.3389/fmed.2024.1478122)
Supplement: Supplementary file 1 [file Data_Sheet_1.docx]

Supplementary Material

## 1 Supplementary Figures

**(A)**


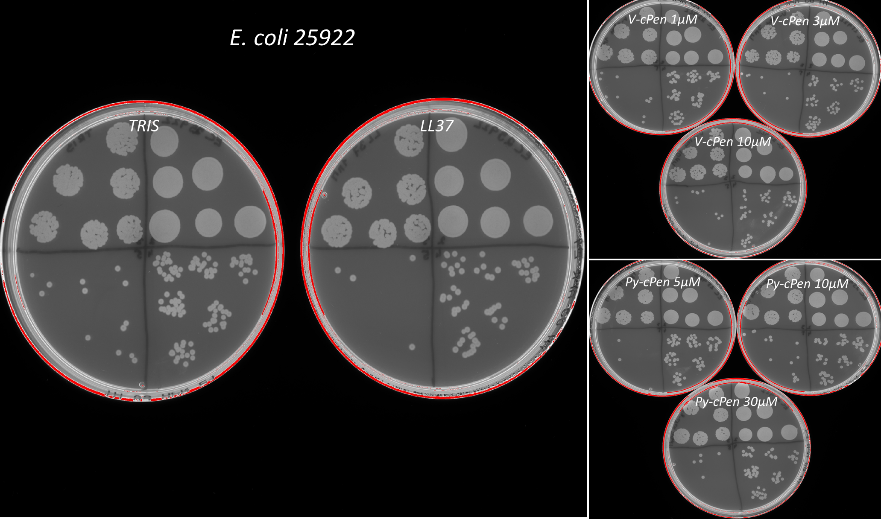


**(B)**


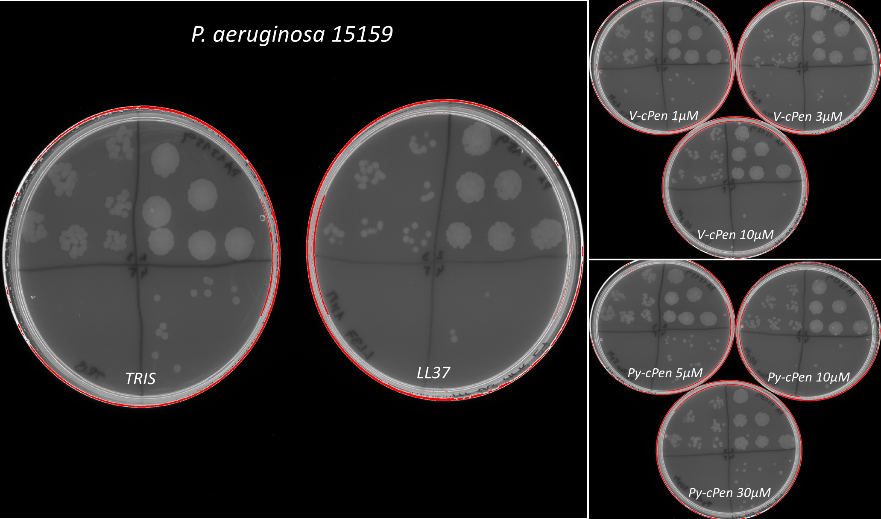


**(C)**


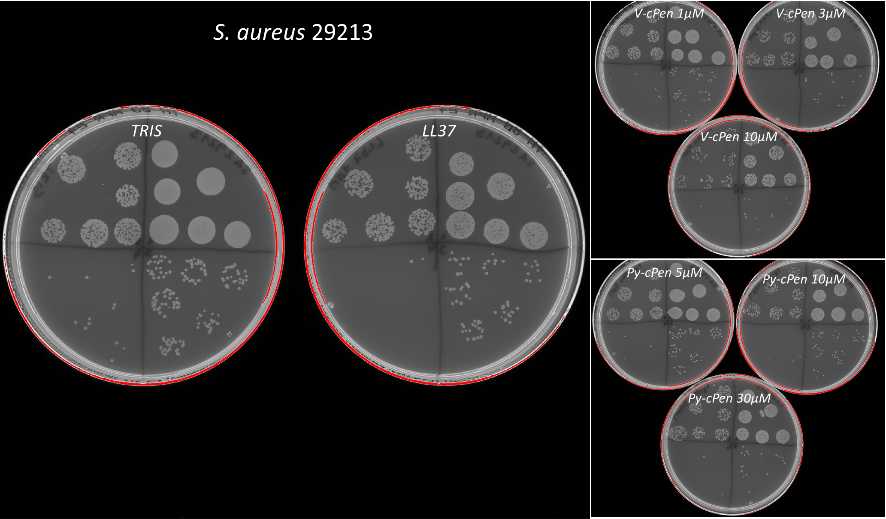


**Supplementary Figure 1**. TH-agar plates were used for analysation of antibacterial effect of Py-cPen (5, 10, 30 µM) and V-cPen (1, 3, 10 µM), LL37 (1 µM) was used as positive control and untreated (using TRIS) bacteria were used as negative control. In the case of E. coli (A), the 4th dilution was used for the calculation, for P. aeruginosa (B) and S. aureus (C) the 3rd dilution was used.

**(A)**


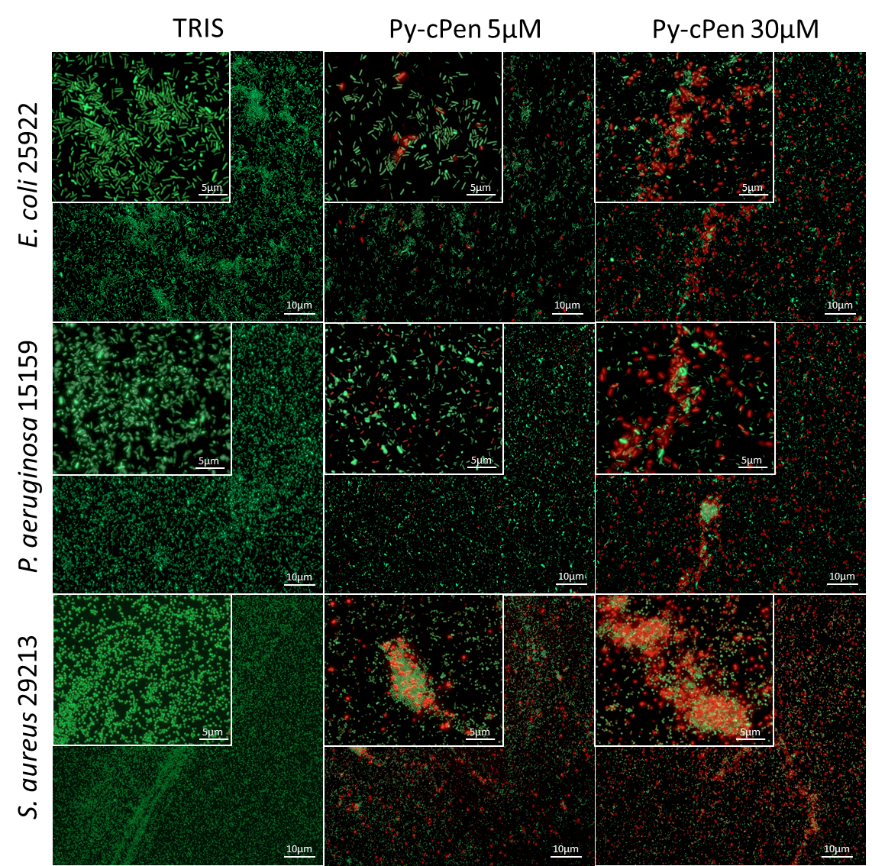


**(B)**

**
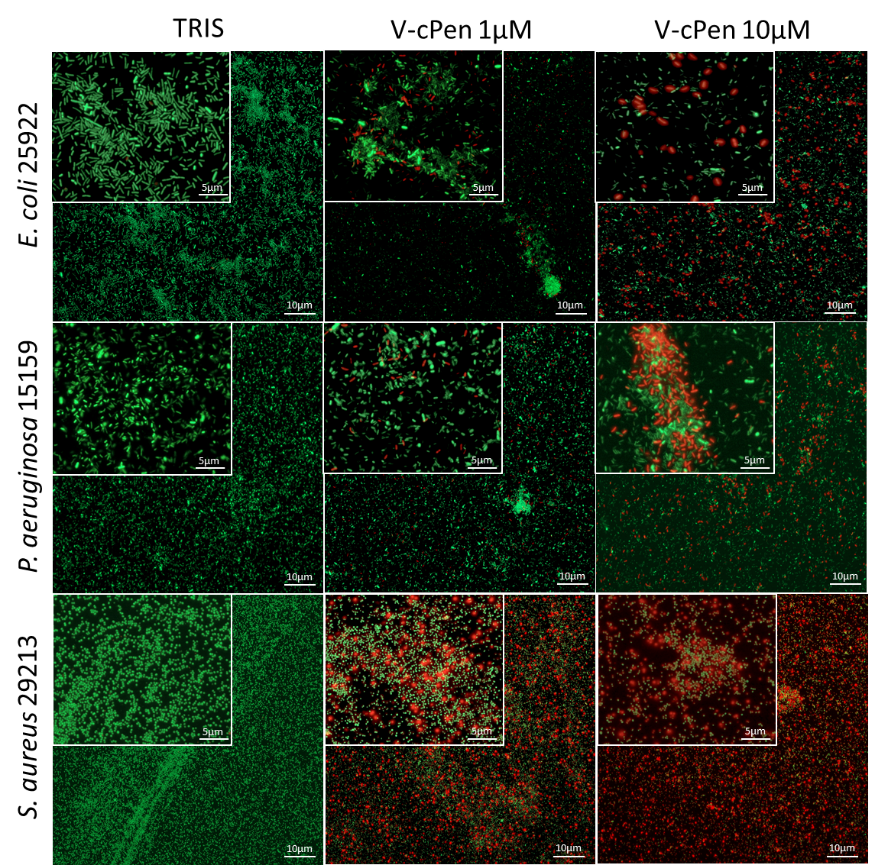
**

**Supplementary figure 2**. Visualization of E. coli, P. aeruginosa and S. aureus viability. Live/Dead Viability Assay of Gram-negative and Gram-positive bacteria stimulated with A) Py-cPen (5 and 30 µM) or B) V-cPen (1 and 10 µM), 10 mM Tris buffer at pH 7.4 as negative control. Live bacteria were stained with green SYTO 9 nucleic acid fluorescent dye, and dead bacteria were stained with red propidium iodine dye.
